# Low-power-consumption CMOS inverter array based on CVD-grown *p*-MoTe_2_ and *n*-MoS_2_

**DOI:** 10.1016/j.isci.2021.103491

**Published:** 2021-11-22

**Authors:** Wanying Du, Xionghui Jia, Zhixuan Cheng, Wanjing Xu, Yanping Li, Lun Dai

**Affiliations:** 1 State Key Lab for Artificial Microstructure & Mesoscopic Physics and Frontiers Science Center for Nano-optoelectronics, School of Physics, Peking University, Beijing 100871, China; 2 Collaborative Innovation Center of Quantum Matter, Beijing 100871, China; 3 Peking University Yangtze Delta Institute of Optoelectronics, Beijing 100871, China

**Keywords:** Engineering, Nanomaterials, Devices

## Abstract

Two-dimensional (2D) semi-conductive transition metal dichalcogenides (TMDCs) have shown advantages for logic application. Complementary metal-oxide-semiconductor (CMOS) inverter is an important component in integrated circuits in view of low power consumption. So far, the performance of the reported TMDCs-based CMOS inverters is not satisfactory. Besides, most of the inverters were made of mechanically exfoliated materials, which hinders their reproducible production and large-scale integration in practical application. In this study, we demonstrate a practical approach to fabricate CMOS inverter arrays using large-area *p*-MoTe_2_ and *n*-MoS_2_, which are grown via chemical vapor deposition method. The current characteristics of the channel materials are balanced by atomic layer depositing Al_2_O_3_. Complete logic swing and clear dynamic switching behavior are observed in the inverters. Especially, ultra-low power consumption of ∼0.37 nW is achieved. Our work paves the way for the application of 2D TMDCs materials in large-scale low-power-consumption logic circuits.

## Highlights

• A practical approach to fabricate large-scale CMOS inverter arrays is demonstrated
• A method to balance the current characteristics of the channel materials is developed
• Complete logic swing and clear dynamic switching behavior are observed
• Ultra-low power consumption of ∼0.37 nW is achieved

## Graphical abstract

**Figure:**
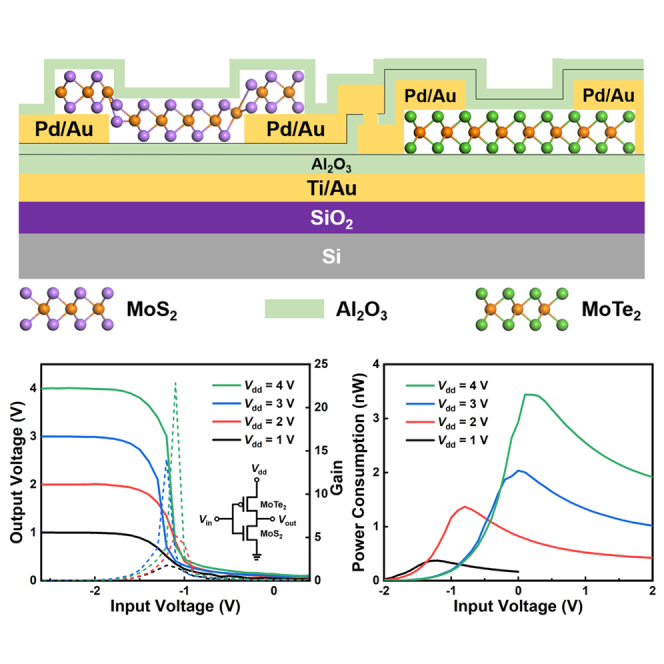

## Introduction

Over the years, two-dimensional (2D) materials such as graphene and transition metal dichalcogenides (TMDCs) have stimulated great research enthusiasm, owing to their unique electronic and optoelectronic properties and ultrathin geometry (Akinwande et al., 2019; Fiori et al., 2014; Liu et al., 2021). Graphene, with high conductivity and high carrier mobility, has been extensively studied (Castro Neto et al., 2009; Flory et al., 2020; Novoselov et al., 2005; Yu et al., 2013). However, because of its gapless nature, graphene is not a good channel material for field-effect transistor (FET), which requires efficient electrostatic control. Semi-conductive TMDCs, with larger bandgaps, surpass graphene in this aspect. Recently, *n*-channel metal-oxide-semiconductor inverters (Wang et al., 2019) and *p*-channel metal-oxide-semiconductor inverters (Zhang et al., 2019) based on TMDCs have been reported. Complementary metal-oxide-semiconductor (CMOS) inverter, composed of an *n*-channel and a *p*-channel FET, has advantage in reducing power consumption and therefore is an important component in integrated circuits. However, so far, the performance of the reported TMDCs-based CMOS inverters is not satisfactory. They suffered from high power consumption (Liu et al., 2019; Pu et al., 2016) or large leakage current (Lin et al., 2014). Besides, most of the CMOS inverters were made of mechanically exfoliated TMDCs (Cho et al., 2019; Jeon et al., 2015; Pezeshki et al., 2016), which hinders their reproducible production and large-scale integration in practical application. Recently, large-area growth in a cost-effective way has been realized for several TMDCs via chemical vapor deposition (CVD) method (Pu et al., 2016; Wang et al., 2019; Xu et al., 2019a). The as-grown MoTe_2_ (Xu et al., 2019b) and MoS_2_ (Wang et al., 2019) are *p*- and *n*-type, respectively. Moreover, it is demonstrated that atomic layer deposition (ALD) of Al_2_O_3_ under certain conditions can cause *n*-type doping to 2D materials, including graphene (Zheng et al., 2015), MoS_2_ (Li et al., 2017), and MoTe_2_ (Lim et al., 2017; Park et al., 2019).

In this study, we fabricate CMOS inverter arrays using large-area CVD-grown *p*-MoTe_2_ and *n*-MoS_2_. We have developed a method to balance the current characteristics of the channel materials. Complete logic swing is obtained in our inverters. High voltage gain (∼23, much larger than 1) and noise margins close to ideal values are obtained. Especially, ultra-low peak power consumption of ∼0.37 nW is achieved, which is among the lowest power consumption values reported so far for TMDCs-based CMOS inverters under similar measurement conditions (Cho et al., 2019; Jeon et al., 2015; Pezeshki et al., 2016). We also investigate the dynamic switching behavior of the CMOS inverters and observe satisfying rising time *t*_r_ (several hundred microseconds) and falling time *t*_f_ (several hundred microseconds). Our work paves the way for the application of 2D TMDCs materials in large-scale low-power-consumption logic circuits.

## Results and discussions

**Figure 1:**
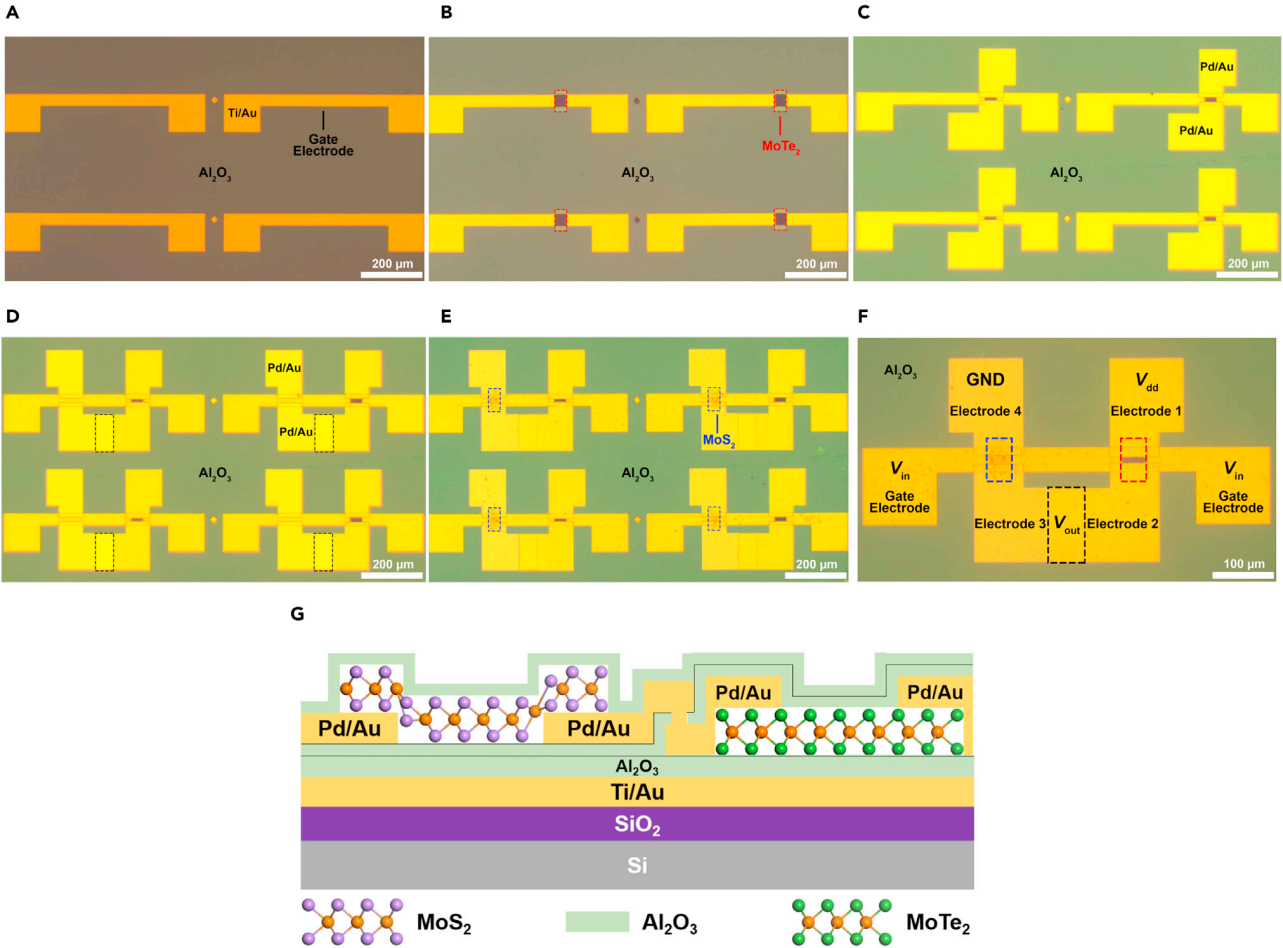
Optical images that illustrate the fabrication steps of the CMOS inverter array (A) Arrayed Ti/Au (10/50 nm) electrodes were fabricated on a SiO_2_/Si substrate as buried gates for both of MoTe_2_ and MoS_2_ FETs. Then a 20-nm-thick Al_2_O_3_ dielectric layer was deposited on the substrate via ALD. (B) A CVD-grown MoTe_2_ film was transferred onto the Al_2_O_3_ layer and patterned into rectangular sheets (outlined by the red dashed lines) over the buried gates. (C) Pairs of Pd/Au (10/50 nm) source and drain electrodes (Electrodes 1 and 2 in (F)) were fabricated on the ends of each MoTe_2_ sheet. After that, a 3-nm-thick Al_2_O_3_ layer was deposited on the whole substrate to protect MoTe_2_ from subsequent steps. (D) Pairs of Pd/Au (10/50 nm) source and drain electrodes (Electrodes 3 and 4 in (F)) were fabricated on the Al_2_O_3_ layer. Herein, Electrode 3 and Electrode 2 have an overlapping area in the vertical direction (outlined by the black dashed lines) for measurement purpose. (E) The MoS_2_ channels (outlined by the blue dashed lines) were fabricated by transferring and patterning a CVD-grown MoS_2_ film. Finally, a 5-nm-thick Al_2_O_3_ layer was deposited on the whole substrate, which caused an *n*-type doping effect on MoS_2_. (F) The optical image of a single inverter in the CMOS inverter array. The red, blue, and black dashed lines outline the MoTe_2_ sheet, the MoS_2_ sheet, and the overlapping area of Electrodes 2 and 3, respectively. To construct a complete CMOS circuit, the buried Gate Electrode and Electrode 1 were connected to *V*_in_ and *V*_dd_, respectively. Electrodes 2 and 3 were connected to a digital oscilloscope by a tungsten needle for *V*_out_ extraction. Electrode 4 was grounded. (G) The cross-sectional schematic of the device structure depicted in (F).

Figures 1A−1E are the corresponding optical images after each fabrication step, illustrating the fabrication process of our CMOS inverter array and demonstrating the feasibility of the large-scale fabrication method. First, arrayed Ti/Au (10/50 nm) electrodes were fabricated on a SiO_2_ (285 nm)/*p*^+^-Si substrate as buried gates for both of MoTe_2_ and MoS_2_ FETs. Then a 20-nm-thick Al_2_O_3_ dielectric layer was deposited on the substrate via ALD method (Figure 1A). Second, a CVD-grown MoTe_2_ film (see Method details) was transferred onto the Al_2_O_3_ layer from the growth substrate with the help of polymethyl methacrylate (PMMA) and deionized water, which avoids the common use of hydrofluoric acid (Pu et al., 2016; Xu et al., 2019b). The transferred MoTe_2_ film was patterned (see Method details) into rectangular sheets (outlined by the red dashed lines) over the buried gates (Figure 1B). Third, pairs of Pd/Au (10/50 nm) source and drain electrodes (Electrodes 1 and 2) were fabricated on the ends of each MoTe_2_ sheet. After that, a 3-nm-thick Al_2_O_3_ layer was deposited on the MoTe_2_ FET array to protect MoTe_2_ from subsequent steps (Figure 1C). For fabricating the MoS_2_ FET array, pairs of Pd/Au (10/50 nm) source and drain electrodes (Electrodes 3 and 4) were fabricated on the Al_2_O_3_ layer (Figure 1D). Herein, Electrode 3 and Electrode 2 have an overlapping area in the vertical direction (outlined by the black dashed lines) for measurement purpose. Similarly, MoS_2_ channels (outlined by the blue dashed lines) were fabricated by transferring and patterning a CVD-grown MoS_2_ film (see Method details). Finally, a 5-nm-thick Al_2_O_3_ layer was deposited on the whole substrate (Figure 1E), which caused an *n*-type doping effect on MoS_2_. Figure 1F is the optical image of a single inverter in the CMOS inverter array. To construct a complete CMOS circuit, the buried Gate Electrode and Electrode 1 were connected to the input voltage (*V*_in_) and supply voltage (*V*_dd_), respectively. Electrodes 2 and 3 were connected to a digital oscilloscope by a tungsten needle for output voltage (*V*_out_) extraction. Electrode 4 was grounded. The cross-sectional schematic of the device structure depicted in Figure 1F is shown in Figure 1G. The CMOS circuit diagram is shown in the inset of Figure 3C. Notably, in this work, we fixed the MoS_2_ FETs' channel length (15 μm) and changed the MoTe_2_ FETs' channel lengths to make their current characteristics balanced. The optical image of the CMOS inverter array is presented in Figure S1C.

**Figure 2:**
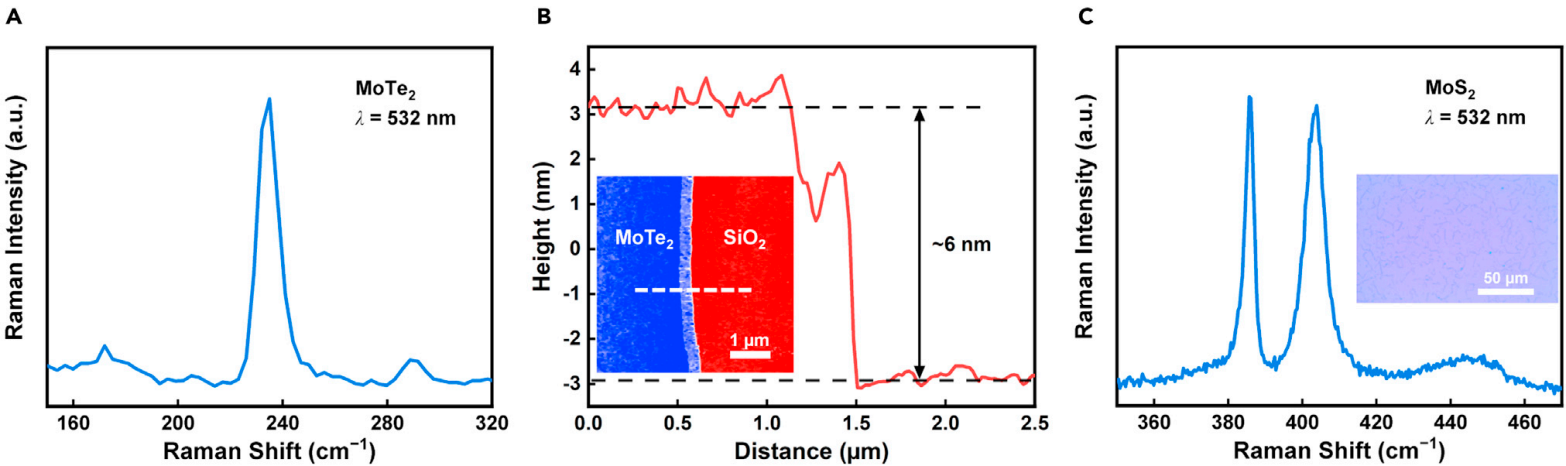
Optical, Raman, and AFM characterizations of the as-grown MoTe_2_ and MoS_2_ (A) The Raman spectrum of the as-grown MoTe_2_ under 532 nm laser illumination, which is dominated by the Raman characteristic peak of 2H-MoTe_2_ at ∼235 cm^−1^. (B) The AFM image of the MoTe_2_ film and the surface height profile along the white dashed line. The MoTe_2_ is about 6 nm thick, corresponding to 9-layer MoTe_2_. (C) The Raman spectrum of the as-grown MoS_2_ under 532 nm laser illumination, which includes the Raman characteristic peaks of MoS_2_ at ∼386 and ∼404 cm^−1^, with peak distance of ∼18 cm^−1^, indicating the monolayer nature of the MoS_2_. Inset: the optical image of the MoS_2_ film. The MoS_2_ film is formed by single crystal MoS_2_ domains.

The MoTe_2_ films (Figure S1A) used in our devices are formed by seamlessly stitched single crystal MoTe_2_ domains (Xu et al., 2019a). Figure 2A shows the Raman spectrum of an as-grown MoTe_2_ film, which presents the Raman characteristic peak of 2H-MoTe_2_ at ∼235 cm^−1^ (Yamamoto et al., 2014). Figure 2B shows the atomic force microscope (AFM) image of the MoTe_2_ and the surface height profile along the white dashed line. The MoTe_2_ is about 6 nm thick, corresponding to 9-layer MoTe_2_ (Lin et al., 2014). The MoS_2_ films (Figure S1B) used in our devices are formed by single crystal MoS_2_ domains (see the inset of Figure 2C). Figure 2C shows the Raman spectrum of an as-grown MoS_2_ film, which presents Raman characteristic peaks of MoS_2_ at ∼386 and ∼404 cm^−1^ (Li et al., 2012). The peak distance is ∼18 cm^−1^, demonstrating the monolayer nature of the MoS_2_ (Li et al., 2012).

**Figure 3:**
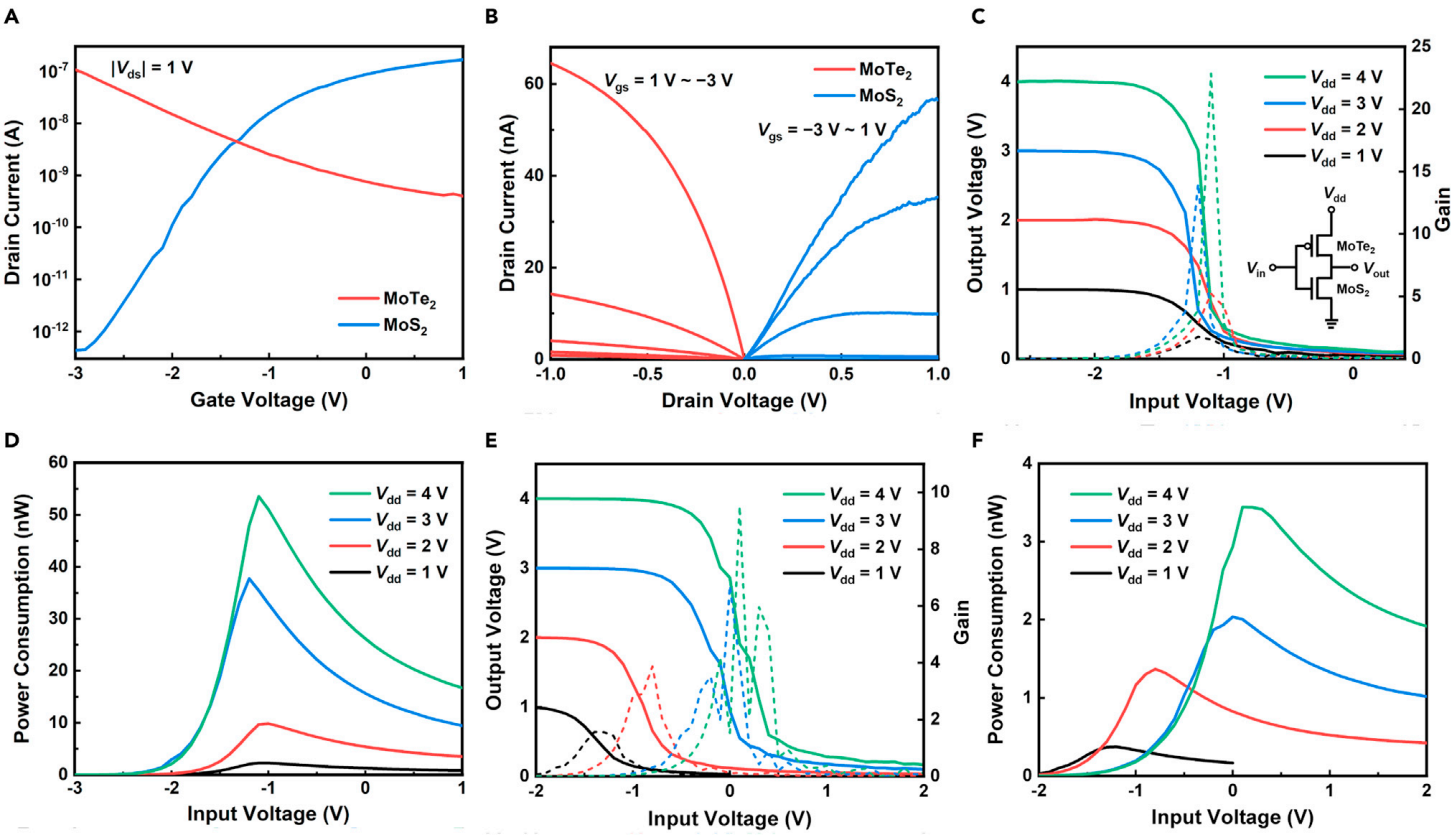
Static operation performance of the inverters (with MoTe_2_ channel length of 15 μm) (A) The transfer curves of the MoTe_2_ and MoS_2_ FETs in an inverter at |*V*_ds_| = 1 V. (B) The output curves of the MoTe_2_ and MoS_2_ FETs in the inverter at various *V*_gs_ with a step of 1 V. (C) The VTCs and voltage gain (−d*V*_out_/d*V*_in_) plots of the inverter at various *V*_dd_. The solid and dashed lines correspond to the output voltage and gain, respectively. Inset: the CMOS circuit diagram. For each *V*_dd_ applied (from 1 to 4 V), the VTC presents complete logic swing, and the maximum voltage gain is bigger than 1. At *V*_dd_ of 4 V, a maximum voltage gain of ∼23 and good noise margins (NM_L_ ≈ 0.40 *V*_dd_, NM_H_ ≈ 0.44 *V*_dd_, total noise margin ≈ 0.84 *V*_dd_) are obtained. (D) The power consumption (*V*_dd_ × *I*_dd_) characteristics of the inverter at various *V*_dd_. At *V*_dd_ of 1 V, peak power consumption of as low as ∼2.3 nW is achieved. (E) The VTCs and voltage gain plots of another inverter at various *V*_dd_. The solid and dashed lines correspond to the output voltage and gain, respectively. For each *V*_dd_ applied (from 1 to 4 V), the VTC presents complete logic swing. At *V*_dd_ of 4 V, a maximum voltage gain of ∼9.5 and good noise margins (NM_L_ ≈ 0.36 *V*_dd_, NM_H_ ≈ 0.40 *V*_dd_, total noise margin ≈ 0.76 *V*_dd_) are obtained. (F) The power consumption characteristics of the inverter corresponding to (E) at various *V*_dd_. At *V*_dd_ of 1 V, ultra-low peak power consumption of ∼0.37 nW is achieved.

Figures 3A and 3B show the transfer curves and output curves of the MoTe_2_ FET (with 15 μm channel length) and MoS_2_ FET in an inverter, respectively. The transfer curves are approximately symmetrical about their intersection. Note that the two ALD steps in the device fabrication process balanced the carrier concentrations of the MoTe_2_ and MoS_2_, enabling the realization of high-performance CMOS inverters (Figure S2). It is worth noting that, in order to avoid the ALD-caused *n*-type doping effect on the *p*-MoTe_2_, we also fabricated a CMOS inverter array with another device structure, where the MoTe_2_ is free from Al_2_O_3_ coverage (Figure S4). The overall device performance did not improve (Figure S5). Figure 3C shows the voltage transfer characteristics (VTCs) and voltage gain (−d*V*_out_/d*V*_in_) plots of the inverter corresponding to Figures 3A and 3B. The inset is the CMOS circuit diagram. For each *V*_dd_ applied (from 1 to 4 V), the VTC presents complete logic swing, and the maximum voltage gain is bigger than 1, satisfying the requirement for logic application. At *V*_dd_ of 4 V, a maximum voltage gain of ∼23 and good noise margins (NM_L_ ≈ 0.40 *V*_dd_, NM_H_ ≈ 0.44 *V*_dd_, total noise margin ≈ 0.84 *V*_dd_) are obtained, indicating the potential of the CMOS inverter to be integrated into complex circuit systems (Wang et al., 2019). The noise margins for high input voltage (NM_H_) and low input voltage (NM_L_) are defined as NM_H_ = *V*_OH_ − *V*_IH_ and NM_L_ = *V*_IL_ – *V*_OL_. *V*_OH_ and *V*_OL_ are the highest and lowest output voltages, respectively. *V*_IH_ and *V*_IL_ are, respectively, the higher and lower input voltages, at which the voltage gains of the VTC equal 1. The total noise margin is the sum of NM_H_ and NM_L_. To evaluate the noise margins of our CMOS inverter, the input voltage range of the VTC is shifted to be symmetric with the output voltage range (Das et al., 2014). Figure 3D shows the power consumption (*V*_dd_ × *I*_dd_) characteristics of the inverter. At *V*_dd_ of 1 V, peak power consumption of as low as ∼2.3 nW is achieved. Figures 3E and 3F show the VTCs and power consumption characteristics of another inverter (with MoTe_2_ channel length of 15 μm). For each *V*_dd_ applied (from 1 to 4 V), the VTC also presents complete logic swing. At *V*_dd_ of 4 V, a voltage gain of ∼9.5 and good noise margins (NM_L_ ≈ 0.36 *V*_dd_, NM_H_ ≈ 0.40 *V*_dd_, total noise margin ≈ 0.76 *V*_dd_) are obtained. Especially, ultra-low peak power consumption of ∼0.37 nW is achieved at *V*_dd_ of 1 V. The statistical gain and power consumption data of the CMOS inverter array are presented in Figures S3A and S3B. It is worth noting that the peak power consumption (0.37–2.3 nW) of our inverters at *V*_dd_ of 1 V is lower compared with previously reported CMOS inverters based on *p*-MoTe_2_ and *n*-MoS_2_ and among the lowest peak power consumption values reported so far for TMDCs-based CMOS inverters (see Table 1). Besides, all the inverters exhibit maximum voltage gains of >1 at each *V*_dd_ applied.

**Table 1:** Power consumption comparison of the CMOS inverters based on TMDCs

Channel materials	*V*_dd_ (V)	Peak power consumption (nW)	Measurement environment	Reference
CVD *p*-MoTe_2_ and *n*-MoS_2_	1	0.37	Dark Ambient	This work
Ex. *p*-MoTe_2_ and *n*-MoS_2_	0.25 1	0.4 3–4	Dark Room temperature	Pezeshki et al. (2016)
Ex. *p*-MoTe_2_ and *n*-MoS_2_	1	4	Dark Room temperature	Cho et al. (2019)
Ex. *p*-MoTe_2_ and *n*-MoS_2_	3	400	Dark Ambient	Seo and Jin (2019)
Ex. *p*-WSe_2_ and *n*-MoS_2_	1 5	0.1–1 1–10	Dark	Jeon et al. (2015)
Ex. *p*-WSe_2_ and *n*-MoS_2_	1	1 × 10^3^	Dark Room temperature	Liu et al. (2019)
CVD *p*-WSe_2_ and *n*-MoS_2_	1	20	N_2_ atmosphere Room temperature	Pu et al. (2016)
Ex. *p*-MoTe_2_ and *n*-MoTe_2_ (*n*-doped)	1	5	Dark Room temperature	Lim et al. (2017)
Ex. *p*-WSe_2_ and *n*-MoS_2_	1.1	0.2	Under 10^−5^ bar	Wang et al., 2020

Ex., mechanically exfoliated; CVD, CVD-grown.

**Figure 4:**
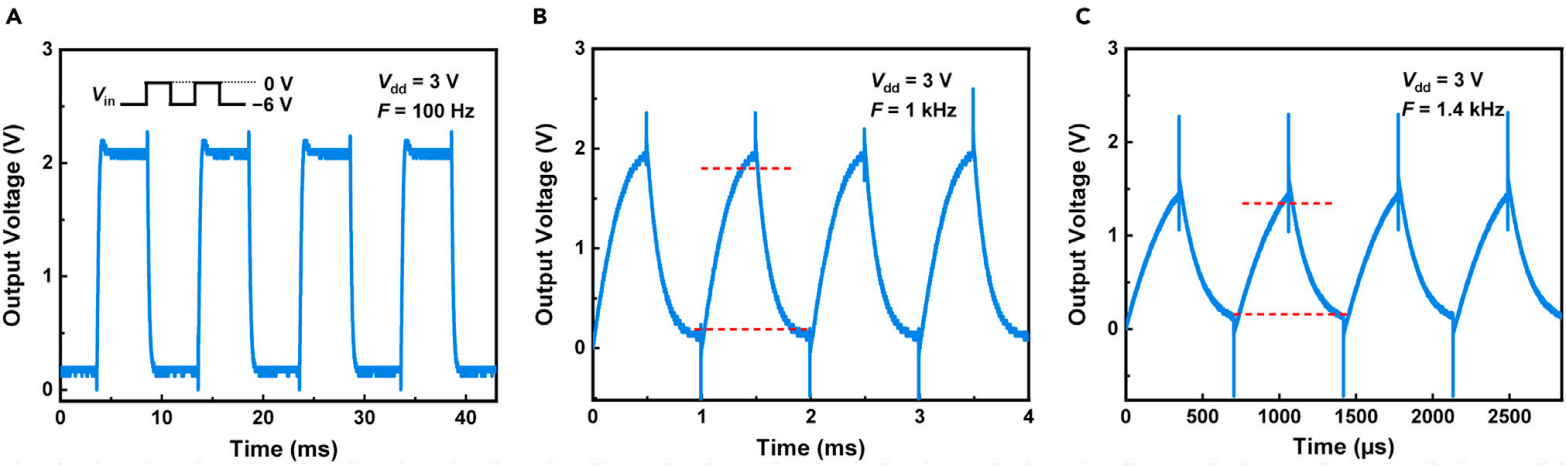
Dynamic switching behavior of an inverter (with MoTe_2_ channel length of 10 μm) (A−C) Time-dependent *V*_out_ at *V*_dd_ of 3 V driven by square wave *V*_in_ with various frequencies. The high and low levels of the input square wave were 0 and −6 V, respectively. Logic switching behavior is clear at 100 Hz and remains to be observed at a critical logic switching frequency of 1 kHz. The rising time (*t*_r_) and falling time (*t*_f_) are about 340 and 308 μs, respectively, at 1 kHz, calculated at 10% and 90% (marked by the red dashed lines in (B)) of *V*_out_ amplitude. At 1.4 kHz, the amplitude of *V*_out_ decreased to half (∼1.5 V) of *V*_dd_, with *t*_r_ (*t*_f_) of about 275 μs (290 μs).

We also investigated the dynamic switching behavior of the CMOS inverters. Figures 4A−4C show the time-dependent *V*_out_ of an inverter (with MoTe_2_ channel length of 10 μm) at *V*_dd_ of 3 V, driven by square wave *V*_in_ with various frequencies. The high and low levels of the input square wave were 0 and −6 V, respectively. Logic switching behavior is clear at 100 Hz and remains to be observed at a critical logic switching frequency of 1 kHz. The *t*_r_ and *t*_f_ are about 340 and 308 μs, respectively, at 1 kHz, calculated at 10% and 90% (marked by the red dashed lines in Figure 4B) of *V*_out_ amplitude. The RC delays mainly result from the overlap capacitance in the CMOS circuit (Pezeshki et al., 2016; Wang et al., 2019). At 1.4 kHz, the amplitude of *V*_out_ decreased to half (∼1.5 V) of *V*_dd_, with *t*_r_ (*t*_f_) of about 275 μs (290 μs). The statistical dynamic switching frequency data of the CMOS inverter array are presented in Figure S3C.

### Conclusions

We have fabricated CMOS inverter arrays using large-area CVD-grown *p*-MoTe_2_ and *n*-MoS_2_. The current characteristics of the channel materials were balanced by atomic layer depositing Al_2_O_3_ under proper conditions. Complete logic swing and clear dynamic switching behavior are observed in the inverters. The inverters have overall high performance such as maximum voltage gains of >1 at each *V*_dd_ applied, low and even ultra-low peak power consumption (0.37–2.3 nW), and satisfying *t*_r_ (*t*_f_) and working frequencies. Our low-power-consumption CMOS inverters, with the merits of reproducibility and large-scale integration, have promising applications in future 2D microelectronic systems.

### Limitation of the study

In order to further improve the device performance, developing new methods to increase the grain size of the monolayer MoS_2_ is needed.

## STAR★Methods

### Key resources table

**Table:** 

REAGENT or RESOURCE	SOURCE	IDENTIFIER
**Chemicals, peptides, and recombinant proteins**		

Tellurium	Zhongnuoxincai	CAS: 13494-80-9
Solid PTAS	2D Semiconductors	N/A
Molybdenum(VI) oxide	Ourchem	CAS: 1313-27-5
Sulfur	Aladdin	CAS: 7704-34-9
PMMA	AllRESIST	CAS: 9011-14-7

### Resource availability

#### Lead contact

Further information and requests for resources should be directed to and will be fulfilled by the lead contact, Lun Dai (lundai@pku.edu.cn).

#### Materials availability

This study did not generate new unique reagents.

### Method details

#### CVD growth of large-area MoTe_2_ and MoS_2_

Large-area MoTe_2_ and MoS_2_ films were grown via CVD method. For MoTe_2_ growth, Mo films were deposited on SiO_2_ (285 nm)/*p*^+^-Si substrates via magnetron sputtering. Then, the substrates were placed in a quartz boat containing Te powder. Molecular sieves were placed in the quartz boat between the substrates and the Te powder. After that, the quartz boat was pushed into the center heating zone of a quartz tube furnace with a tube diameter of 1 inch. After evacuating the quartz tube to an air pressure of less than 1 mTorr, high-purity Ar was let in at the maximum flow rate until the pressure reached atmospheric pressure. Next, the furnace was heated to 650°C in 30 min and kept there for 180 min. High-purity H_2_ and Ar were used as carrier gases, whose flow rates were 7 and 5 standard cubic centimeters per minute (sccm), respectively. After the growth, the furnace cooled to room temperature naturally. For MoS_2_ growth, SiO_2_ (285 nm)/*p*^+^-Si substrates were processed with O_2_ plasma. After that, perylene-3,4,9,10-tetracarboxylic acid tetrapotassium salt (PTAS) was spin-coated on the substrates as seeding promoter. The substrates were placed in a quartz boat containing MoO_3_ powder. Another quartz boat with S power was pushed into the upstream heating zone of a 3-temperature-zone quartz tube furnace with a tube diameter of 2 inch. Then the quartz boat with growth substrates was pushed into the downstream heating zone of the furnace. The growth was performed at atmospheric pressure. High-purity Ar (15 sccm) was used as carrier gas. The upstream and downstream heating zones of the furnace were heated to 200°C and 650°C, respectively, in 40 min, and kept there for 5 min. Finally, the furnace cooled to room temperature naturally.

#### Fabrication of the CMOS inverter arrays

Both the CVD-grown MoTe_2_ and MoS_2_ films were transferred with the help of PMMA and deionized water (Kim et al., 2019), and patterned into rectangular sheets through ultra-violet (UV) lithography and reactive ion etching. The Ti/Au and Pd/Au electrodes were fabricated via UV lithography, electron beam evaporation, and lift-off process. The Al_2_O_3_ layers were deposited using an ALD system (Cambridge NanoTech Inc., Savannah-100). The patterned Al_2_O_3_ layer (see Figure S4F) was fabricated via UV lithography, ALD, and lift-off process. In the ALD process, trimethylaluminum and deionized water served as precursors and high-purity N_2_ served as carrier gas. The reaction temperatures were 200°C for Al_2_O_3_ films covering the whole substrate and 80°C for the patterned Al_2_O_3_ layer (because the photoresist could not endure the temperature of 200°C). The Al_2_O_3_ thickness was controlled by deposition time.

#### Characterizations

The optical images of the devices were taken by an optical microscope (ZEISS, Axio Imager A2m). The Raman spectra were collected by a micro-zone confocal Raman system (WITec alpha 300R) under 532 nm laser illumination. The thickness of the MoTe_2_ film was measured by an atomic force microscope (Asylum Research, Cypher S). All the electrical measurement was conducted in the dark with a semiconductor characterization system (Keithley 4200-SCS) that was connected to a probe station. For the dynamic switching performance measurement, a function generator (Tektronix AFG 3102) and a digital oscilloscope (Tektronix DPO 2024) were employed to generate the *V*_in_ and record the *V*_out_, respectively. Both the function generator and the digital oscilloscope had common ground with the semiconductor characterization system, which provided the *V*_dd_. All the characterizations were performed in ambient condition.

## Data Availability

All data reported in this paper will be shared by the lead contact upon request. This paper does not report original code. Any additional information required to reanalyze the data reported in this paper is available from the lead contact upon request.
