# Supplementary material for: Low-power-consumption CMOS inverter array based on CVD-grown p-MoTe2 and n-MoS2
Source: iScience. 2021 Nov 22;24(12):103491. doi: 10.1016/j.isci.2021.103491 (PMC8668989; doi:10.1016/j.isci.2021.103491)
Supplement: Document S1. Figures S1–S5 [file mmc1.pdf]

**iScience, Volume *24***

## Supplemental information

# Low-power-consumption CMOS inverter array based on CVD-grown *p*-$MoTe_2$ and *n*-$MoS_2$

**Wanying Du, Xionghui Jia, Zhixuan Cheng, Wanjing Xu, Yanping Li, and Lun Dai**

# Supplemental Figures

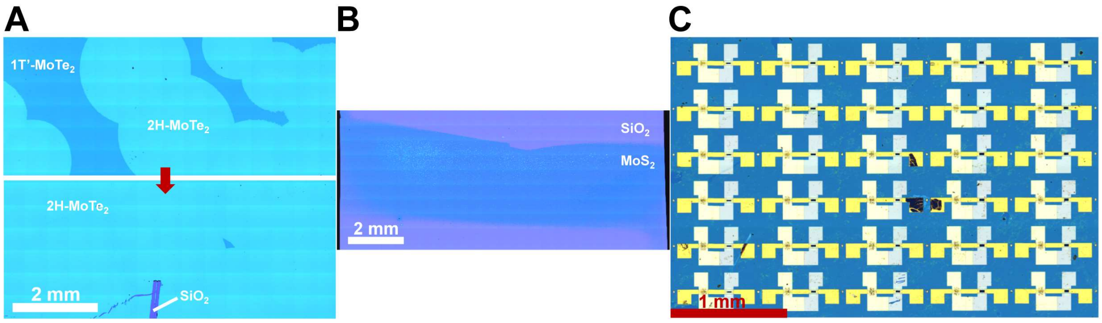


**Figure S1. Optical images of the large-area $MoTe_2$ film, the large-area $MoS_2$ film, and the 5 × 6 CMOS inverter array, related to Figure 1 and Figure 2.**

(A) Optical image of the large-area $MoTe_2$ film. As the growth time increases, the 1T′-$MoTe_2$ gradually phase-transforms into 2H-$MoTe_2$ (the upper image in Figure S1A). After growth, the large-area 2H-$MoTe_2$ film is uniform on the whole substrate (the lower image in Figure S1A). The intentionally scratched area is aimed to demonstrate the different colors between the 2H-$MoTe_2$ film and the $SiO_2$ substrate.

(B) Optical image of the large-area $MoS_2$ film. The colors of the monolayer and multilayer $MoS_2$ are navy-blue and light-blue, respectively.

(C) Optical image of the 5 × 6 CMOS inverter array. In the CMOS inverter array, the $MoS_2$ FETs have only one channel length (15 μm), and the $MoTe_2$ FETs have different channel lengths.

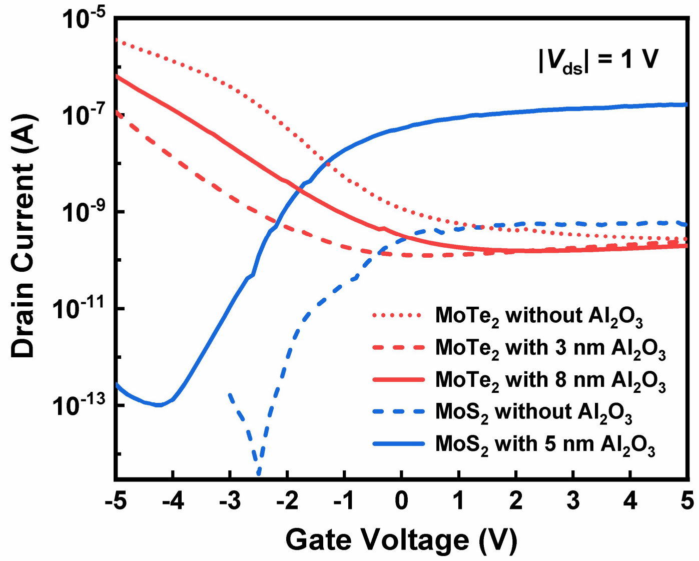


**Figure S2. Transfer curves of the $MoTe_2$ and $MoS_2$ FETs (both with 15 μm channel lengths) measured during the device fabrication process, related to Figure 1 and Figure 3.**

Usually, an $Al_2O_3$ ALD process will cause an *n*-type doping effect on both $MoTe_2$ (Park et al., 2019) and $MoS_2$ (Li et al., 2017). For $MoTe_2$, after the first ALD process (3 nm thick), the hole concentration decreased from $2.45 \times 10^{12}$ $cm^{-2}$ to $1.32 \times 10^{12}$ $cm^{-2}$. However, the hole concentration increased to $1.37 \times 10^{12}$ $cm^{-2}$ after the second ALD process. This indicates that the second ALD process (5 nm thick) would weaken the *n*-type doping effect of the first ALD

process. One possible explanation is that the annealing effect in the second ALD process may counteract the *n*-type doping effect of the first ALD process (Zheng et al., 2015). For $MoS_2$, which experienced only one ALD process (5 nm thick), the electron concentration increased from $1.98 \times 10^{12}$ cm$^{-2}$ to $3.86 \times 10^{12}$ cm$^{-2}$ after the ALD process. Therefore, by intentionally adopting the two ALD processes for $MoTe_2$ FET and one ALD process for $MoS_2$ FET, we balanced the hole concentration of the $MoTe_2$ FET and the electron concentration of the $MoS_2$ FET. As a result, an intersection appeared at the transfer curves of the $MoTe_2$ and $MoS_2$ FETs in the gate voltage range around 0 V, which satisfied the requirement of high-performance CMOS inverter.

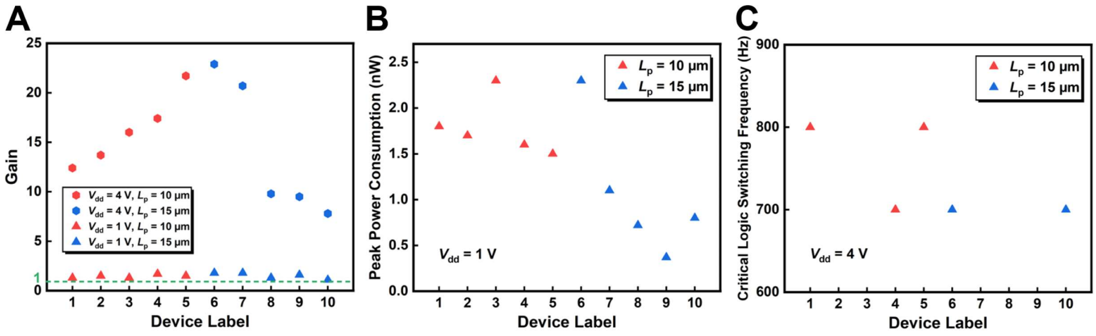


**Figure S3. Statistical data of the CMOS inverter array, related to Figure 3 and Figure 4.**

(A) Statistical data of maximum voltage gains. $L_p$: $MoTe_2$ channel length.

(B) Statistical data of peak power consumption.

(C) Statistical data of critical logic switching frequencies.

At $V_{dd}$ of 1 V, all of the measured inverters show maximum voltage gains of >1 (Figure S3A) and low peak power consumption (0.37−2.3 nW) (Figure S3B). At $V_{dd}$ of 4 V, most of the measured inverters show maximum voltage gains of >10 (Figure S3A) and critical logic switching frequencies around 700−800 Hz (Figure S3C).

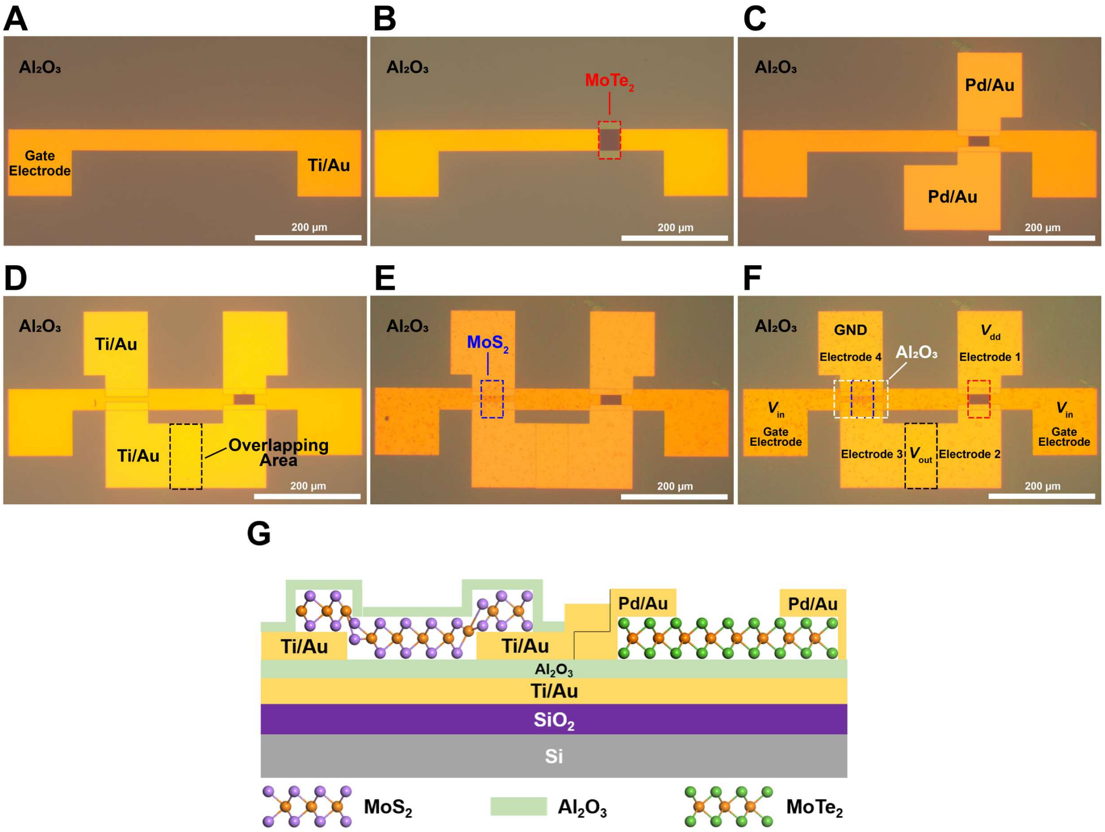


**Figure S4. Optical images that illustrate the fabrication steps of the CMOS inverter array with the other device structure, related to Figure 1 and Figure 3.**

(A) Arrayed Ti/Au (10/50 nm) electrodes were fabricated on a $SiO_2$/Si substrate as buried gates for both of $MoTe_2$ and $MoS_2$ FETs. Then a 20 nm thick $Al_2O_3$ dielectric layer was deposited on the substrate via ALD.

(B) A CVD-grown $MoTe_2$ film was transferred onto the $Al_2O_3$ layer and patterned into rectangular sheets (outlined by the red dashed line) over the buried gates.

(C) Pairs of Pd/Au (10/50 nm) source and drain electrodes (Electrodes 1 and 2 in (F)) were fabricated on the ends of each $MoTe_2$ sheet.

(D) Ti/Au (10/50 nm) source and drain electrodes (Electrodes 3 and 4 in (F)) were fabricated. Herein, Electrode 3 has an overlapping area with Electrode 2 in the vertical direction (outlined by the black dashed line), as required by the CMOS circuit.

(E) The $MoS_2$ channels (outlined by the blue dashed line) were fabricated by transferring and patterning a CVD-grown $MoS_2$ film. The $MoS_2$ FETs have common buried gates with $MoTe_2$ FETs.

(F) $Al_2O_3$ patterns (10 nm thick, outlined by the white dashed line) were fabricated on the $MoS_2$ channels to implement *n*-type doping locally.

(G) The corresponding cross-sectional schematic.

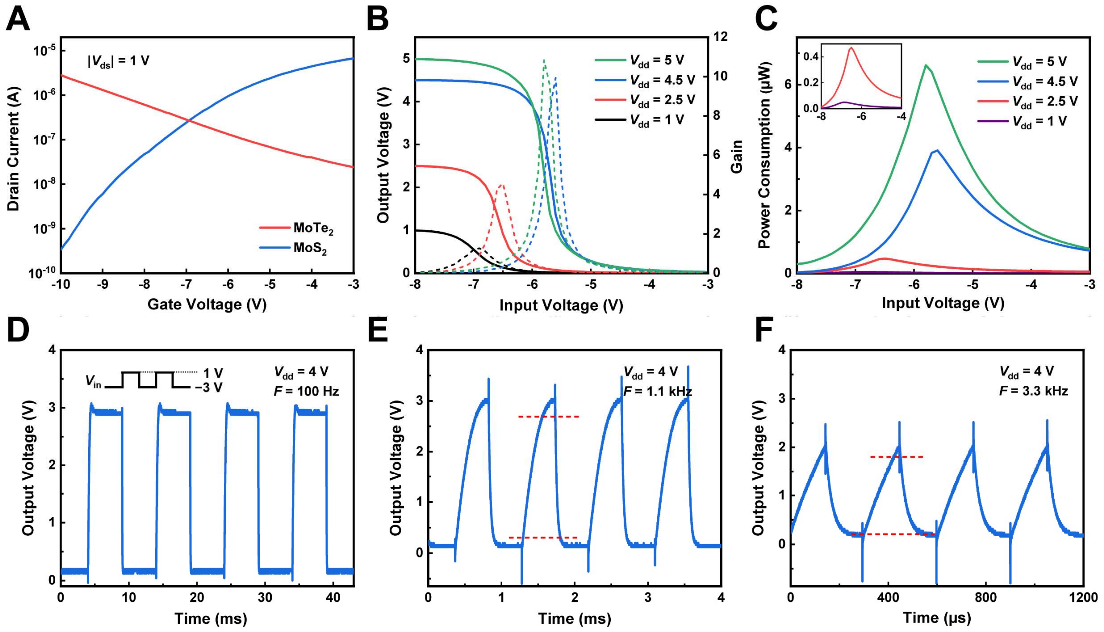


**Figure S5. Static operation performance and dynamic switching behavior of two inverters with the device structure depicted in Figure S4, related to Figure 3 and Figure 4.**

(A) The transfer curves of the $MoTe_2$ FET (with 20 μm channel length) and $MoS_2$ FET (with 10 μm channel length) in an inverter at $|V_{ds}|$ = 1 V.

(B) The VTCs and voltage gain plots of the inverter at various $V_{dd}$. The solid and dashed lines correspond to the output voltage and gain, respectively. At $V_{dd}$ of 5 V, a maximum voltage gain of ~11 and good noise margins ($NM_L \approx 0.42$ $V_{dd}$, $NM_H \approx 0.38$ $V_{dd}$, total noise margin ≈ 0.80 $V_{dd}$) are obtained.

(C) The power consumption characteristics of the inverter at various $V_{dd}$. At $V_{dd}$ of 1 V, peak power consumption of ~49.4 nW is achieved.

(D−F) Time dependent $V_{out}$ of another inverter (with the same channel lengths) at $V_{dd}$ of 4 V driven by square wave $V_{in}$ with various frequencies. The high and low levels of the input square wave were 1 and −3 V, respectively. Shorter $t_r$ ($t_f$) of about 265 μs (67 μs) is observed at 1.1 kHz. Besides, the amplitude of $V_{out}$ decreased to half (~2 V) of $V_{dd}$ at a higher $V_{in}$ frequency of 3.3 kHz, with $t_r$ ($t_f$) of about 122 μs (101 μs).
